# Depression, distress and their association with patterns of psychoactive substance use during the COVID-19 emergency state in latvia

**DOI:** 10.1192/j.eurpsy.2021.654

**Published:** 2021-08-13

**Authors:** J. Vrublevska, A. Šibalova, I. Aleskere, B. Rezgale, D. Smirnova, K. Fountoulakis, E. Rancans

**Affiliations:** 1 Department Of Psychiatry And Narcology, Riga Stradins University, Riga, Latvia; 2 Faculty Of Residency, Riga Stradins University, Riga, Latvia; 3 Department Of Psychiatry, Narcology, Psychotherapy And Clinical Psychology, Samara State Medical University, Samara, Russian Federation; 4 3rd Department Of Psychiatry, School Of Medicine, Aristotle University of Thessaloniki, Thessaloniki, Greece

**Keywords:** Co-morbidities, Depression, Alcohol, COVID-19

## Abstract

**Introduction:**

The WHO warned that the COVID-19 pandemic could have psychiatric consequences such as elevated levels depression, increased alcohol and drug use, and other behaviours that exert a strong influence on health. In Latvia a state of emergency was announced on March 12^th^, was extended twice and lifted on June 10th.

**Objectives:**

To estimate the prevalence of depression and distress in the general population of Latvia and association with substance use during the state of emergency.

**Methods:**

The nationwide representative online study in the general population of Latvia was conducted in July 2020 during three week period. The Center for Epidemiologic Studies Depression Scale (CES-D) was used to determine the presence of distress/depression. The structured questionnaire to determine psychoactive substance use was applied. Proportions of independent variables across the study groups were compared using Chi-square test.

**Results:**

The study sample included 2608 respondents. The prevalence of depression and distress was estimated at 5.7% (95%CI 4.92 – 6.71) and 13.5% (95%CI 6.85 – 8.91), respectively. Patients with depression (28% vs. 7.4%, p <0.001) and distress (30.9% vs. 7.4% p <0.05) smoked more tobacco compared to respondents without distress/depression. Those with depression or distress were significantly more likely to consume more alcohol during the emergency state than people without depression or distress (14.0% and 17.7% vs. 6.6%, p <0.001). The changes in the use of other psychoactive substances in those who had depression or distress were not statistically significant.

**Conclusions:**

Preparing support systems to mitigate mental health consequences is needed urgently.

